# Factors that Influence the Performance of Elite Sprint Cross-Country Skiers

**DOI:** 10.1007/s40279-016-0573-2

**Published:** 2016-06-22

**Authors:** Kim Hébert-Losier, Christoph Zinner, Simon Platt, Thomas Stöggl, Hans-Christer Holmberg

**Affiliations:** 1 Department of Sports Science, National Sports Institute of Malaysia, National Sports Complex, 57000 Bukit Jalil, Kuala Lumpur Malaysia; 2 Department of Health Sciences, Swedish Winter Sports Research Centre, Mid Sweden University, Östersund, Sweden; 3 Department of Sport Science, Julius-Maximilians-University Würzburg, Würzburg, Germany; 4 Department of Sport Science and Kinesiology, University of Salzburg, Salzburg, Austria

## Abstract

**Background:**

Sprint events in cross-country skiing are unique not only with respect to their length (0.8–1.8 km), but also in involving four high-intensity heats of ~3 min in duration, separated by a relatively short recovery period (15–60 min).

**Objective:**

Our aim was to systematically review the scientific literature to identify factors related to the performance of elite sprint cross-country skiers.

**Methods:**

Four electronic databases were searched using relevant medical subject headings and keywords, as were reference lists, relevant journals, and key authors in the field. Only original research articles addressing physiology, biomechanics, anthropometry, or neuromuscular characteristics and elite sprint cross-country skiers and performance outcomes were included. All articles meeting inclusion criteria were quality assessed. Data were extracted from each article using a standardized form and subsequently summarized.

**Results:**

Thirty-one articles met the criteria for inclusion, were reviewed, and scored an average of 66 ± 7 % (range 56–78 %) upon quality assessment. All articles except for two were quasi-experimental, and only one had a fully-experimental research design. In total, articles comprised 567 subjects (74 % male), with only nine articles explicitly reporting their skiers’ sprint International Skiing Federation points (weighted mean 116 ± 78). A similar number of articles addressed skating and classical techniques, with more than half of the investigations involving roller-skiing assessments under laboratory conditions. A range of physiological, biomechanical, anthropometric, and neuromuscular characteristics was reported to relate to sprint skiing performance. Both aerobic and anaerobic capacities are important qualities, with the anaerobic system suggested to contribute more to the performance during the first of repeated heats; and the aerobic system during subsequent heats. A capacity for high speed in all the following instances is important for the performance of sprint cross-country skiers: at the start of the race, at any given point when required (e.g., when being challenged by a competitor), and in the final section of each heat. Although high skiing speed is suggested to rely primarily on high cycle rates, longer cycle lengths are commonly observed in faster skiers. In addition, faster skiers rely on different technical strategies when approaching peak speeds, employ more effective techniques, and use better coordinated movements to optimize generation of propulsive force from the resultant ski and pole forces. Strong uphill technique is critical to race performance since uphill segments are the most influential on race outcomes. A certain strength level is required, although more does not necessarily translate to superior sprint skiing performance, and sufficient strength-endurance capacities are also of importance to minimize the impact and accumulation of fatigue during repeated heats. Lastly, higher lean mass does appear to benefit sprint skiers’ performance, with no clear advantage conferred via body height and mass.

**Limitations:**

Generalization of findings from one study to the next is challenging considering the array of experimental tasks, variables defining performance, fundamental differences between skiing techniques, and evolution of sprint skiing competitions. Although laboratory-based measures can effectively assess on-snow skiing performance, conclusions drawn from roller-skiing investigations might not fully apply to on-snow skiing performance. A low number of subjects were females (only 17 %), warranting further studies to better understand this population. Lastly, more training studies involving high-level elite sprint skiers and investigations pertaining to the ability of skiers to maintain high-sprint speeds at the end of races are recommended to assist in understanding and improving high-level sprint skiing performance, and resilience to fatigue.

**Conclusions:**

Successful sprint cross-country skiing involves well-developed aerobic and anaerobic capacities, high speed abilities, effective biomechanical techniques, and the ability to develop high forces rapidly. A certain level of strength is required, particularly ski-specific strength, as well as the ability to withstand fatigue across the repeated heats of sprint races. Cross-country sprint skiing is demonstrably a demanding and complex sport, where high-performance skiers need to simultaneously address physiological, biomechanical, anthropometric, and neuromuscular aspects to ensure success.

## Key Points

**Table:** 

The structure of sprint cross-country skiing events is quite unique, as it involves four high-intensity heats (each ~3 min in duration) separated by a relatively short recovery period (15–60 min).
Numerous physiological, biomechanical, anthropometric, and neuromuscular factors exert an impact on sprint skiing performance. The key factors that promote good performance include well-developed aerobic and anaerobic capacities, adequate strength and ski-specific power, a high proportion of lean mass, effective skiing biomechanics, and an ability to attain and maintain high speeds during a single heat, as well as a series of heats.

## Introduction

**Fig. 1:**
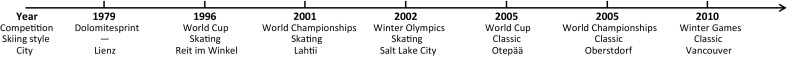
A brief history of sprint cross-country skiing

Cross-country skiing has been contested at the Olympics since the first Winter Games held in Chamonix, France, in 1924. Since then, the sport of cross-country skiing has evolved to include two distinct styles (skating and classic) and a range of race distances (from sprint to long distance events of 800 m to 50 km in length). The Dolomitensprint, featured in Lienz in 1979, is claimed to be the first sprint cross-country skiing race (http://www.dolomitensport.at). Sprint skate skiing races were first introduced officially into the World Cup in 1996 in Reit im Winkl and into World Championships contests in 2001 in Lahti. In 2005, sprint classic skiing sprints appeared in these contests in Otepää and Oberstdorf, respectively (Fig. 1). Sprint events became officially part of the Winter Games for the first time in Salt Lake City in 2002. The most recent 2014 Winter Games in Sochi involved a total of 12 cross-country skiing events (six for men and six for women), of which four were sprint races.

Over the years, several reviews have summarized the evidence relating to the biomechanics [1, 2], physiology [3, 4], and injuries [5, 6] associated with cross-country skiing. However, these reviews do not encompass the scientific literature relating to sprint events. Recently, Sandbakk and Holmberg [7] provided a short invited commentary on factors leading to success in Olympic cross-country skiing in which select similarities and differences between long distance and sprint skiers were highlighted. A number of skiers are able to compete successfully in both distance and sprint events [8]. For example, Marit Bjørgen (Norway) finished first overall in both the distance and sprint events for women during the 2014–2015 World Cup; and Petter Northug (Norway) finished first in both the 50-km and individual-sprint events for men during the 2015 International Ski Federation (FIS) Nordic World Ski Championships. However, although some biomechanical and physiological factors are crucial for high-level performance in both short- and long-distance races, a more precise investigation of factors relating to sprint cross-country skiing performance is needed.

Sprint races are from 1.0 to 1.8 km in length for men and from 0.8 to 1.6 km for women, and are contested in both classic and skating techniques. Sprint races are unique in cross-country skiing not only in terms of length, but also in terms of involving repeated heats. In contrast to most skiing events which have one mass start, sprint races begin with an individual prologue (i.e., time-trial qualification round) from which the 30 fastest skiers progress to knock-out heats. Six athletes compete head-to-head in each heat, with the fastest two skiers from each heat (five heats × two skiers) progressing to the semi-finals along with the two other fastest skiers (lucky losers) who did not finish among the top two of their respective heats. A total of 12 skiers then compete in two semi-final heats comprised of six skiers each, with only six skiers making the finals. Hence, a skier must compete in an individual prologue, a quarterfinal, and a semi-final before reaching the final to have a chance at medalling [9]. This structure makes sprint events quite different from the more traditional distance races, involving four high-intensity heats (each typically ~3 min in duration) separated by a relatively short period of recovery (15–60 min).

At the elite level, sprint cross-country skiing events are extremely competitive. Improvements as small as 0.39 % in sprint cross-country skiing race times have been proposed to represent worthwhile enhancements in prologue performance, which could improve an athlete’s chance of securing a place on the winners’ podium [10]. In parallel, a substantial body of research has emerged regarding sprint cross-country skiing performance over the last decade. For example, investigations have been conducted using a variety of methods, ranging from purely laboratory-based [11–13] to on snow [14, 15] and in competition [16, 17]. These studies have highlighted numerous factors underpinning sprint cross-country skiing performance, which broadly fall under physiology, biomechanics, anthropometry, and neuromuscular. Both aerobic [18] and anaerobic [11] capacities appear to be requisite for high-level performance in sprint skiing, together with the ability to generate high forces [12], select appropriate skiing techniques [15], and utilize optimal skiing biomechanics [12]. It is noteworthy that peak skiing speed has been correlated to performance during a simulated competition involving three 1,100-m heats [19], as well as to skiers’ FIS-sprint points [18]. Given the small winning margins in elite sprint cross-country skiing, there is a need for an in-depth understanding of the factors influencing performance and a detailed appraisal of the literature relating to this particular sport.

In this context, the present systematic review aims to identify and summarize the physiological, biomechanical, anthropometric, and neuromuscular factors that relate to elite sprint cross-country skiing performance. This appraisal should provide an overview of pertinent factors to optimize performance of sprint skiers, as well as serve as a guide for future research in sprint cross-country skiing.

## Methods

This systematic review of the literature adheres to the structure and reporting guidelines of PRISMA (Preferred Reporting Items for Systematic Reviews and Meta-Analyses) [20].

### Search Strategy

**Fig. 2:**
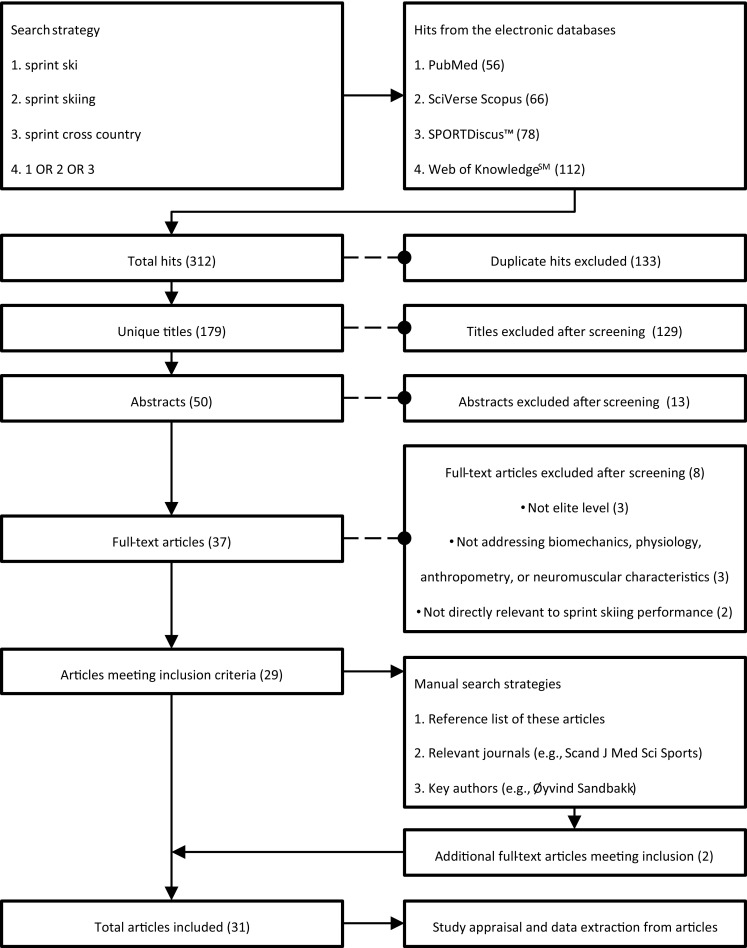
Flow diagram of the search strategy and the article selection process

The PubMed, SciVerse Scopus, SPORTDiscus™, and Web of Knowledge^SM^ databases were searched systematically on 29 March 2015, using “sprint ski OR sprint skiing OR sprint cross country” as the search strategy (Fig. 2). In addition, the reference lists of all articles thus identified and subsequently chosen for inclusion were searched manually for additional articles of relevance, as were publications by key researchers in this field (e.g., Hans-Christer Holmberg, Jussi Mikkola, Thomas Stöggl, Øyvind Sandbakk, and Raphael Zory) and the table of contents of relevant journals (e.g., the Scandinavian Journal of Medicine and Science in Sports, and the International Journal of Sports Physiology and Performance).

### Inclusion and Exclusion Criteria

Only original research articles addressing biomechanics, physiology, anthropometry, or neuromuscular characteristics in combination with sprint cross-country skiing performance were included, whereas articles addressing equipment, environmental, or other external factors were excluded. More precisely, only original research studies that involved elite cross-country skiers (i.e., at least at the national level), assessed sprint skiing performance (either on snow or in the laboratory), related variables to sprint skiing performance, and were published in the English language in peer-reviewed journals were included. Articles that examined peak skiing speed were included only if of direct relevance to sprint skiers and sprint skiing performance at an elite level. Articles that addressed peak skiing speed without contextualization to sprint skiing or elite athletes were excluded. Articles on other types of skiing (e.g., biathlon, distance cross-country skiing, and alpine skiing) and letters to the editor, symposium reports, conference abstracts, special technical publications, books, expert opinions, commentaries, and literature reviews were excluded.

### Study Selection Process

Duplicate articles identified electronically through the different databases searched were removed first. Next, to minimize bias, a third party eliminated any potentially identifiable information (i.e., authors, names, affiliations, country of origin, and journal title). Thereafter, two independent reviewers (CZ and SP) screened all of the titles, abstracts, and full-texts in that order for inclusion and exclusion. Results from the two independent screenings were compared and, in case of disagreement, a third reviewer (KHL) was consulted to reconcile differences. The study selection process was repeated for articles identified through other searches until no additional publications of interest were found.

### Study Appraisal

To assess the quality of articles fulfilling the criteria for inclusion, we employed a modified version of the Downs and Black Quality Assessment Checklist [21], which provides an overall quality score for different study designs and is suitable for articles on elite sport performance [22, 23]. Furthermore, this checklist exhibits high internal consistency (Kuder–Richardson 20 = 0.89), test-retest reliability (*r* = 0.88), inter-rater reliability (*r* = 0.75), and criterion validity in comparison to global scores from the Standards of Reporting Trials Group (*r* *=* 0.90) [21, 24].

Modifications made to the original checklist included replacing the words “patient” with “subject/participant”, “interventions” with “conditions”, and “treatment” with “testing”. On questions 8, 9, 14, 15, 17, 19, 23, 24, and 26, “Not applicable” was added as a fourth scoring option. Question 27 was scored “Yes” (1 point–statistical significance attained), “No” (0 point–no statistical significance), or “Not applicable” (no statistical analyses performed). When an article reported or provided a reference to the accuracy of a measurement system, question 20 was scored “Yes”.

In questions 5 and 25, FIS points, age, and sex were considered to be core confounders; while body mass, specialization (i.e., sprint or distance skiing), country of origin, and years of experience were considered to be other confounders. To receive two points on question 5, all three core confounders and at least one other confounder had to be reported. For one point, three confounders, including at least two core confounders had to be recorded. Otherwise, a score of zero was given. After excluding questions scored as “Not applicable”, the final score was calculated as a percentage: [(total number of points/total number of applicable points) × 100 %], where a higher percentage score indicates a study of superior quality.

Since the quality score did not depend on study design, standard classification schemes [25, 26] were employed to classify the design of each study, first as experimental, quasi-experimental, or non-experimental, and then as a case study, case series, or repeated-measures design. No article was excluded on the basis of its quality score or study design.

The same two investigators (CZ and SP) who screened for inclusion criteria assessed the quality and classified the design of all articles independently. Again, a third reviewer (KHL) reconciled any disagreements, with any potentially identifying information still lacking at this stage from articles.

### Data Extraction, Synthesis, and Analysis

**Fig. 3:**
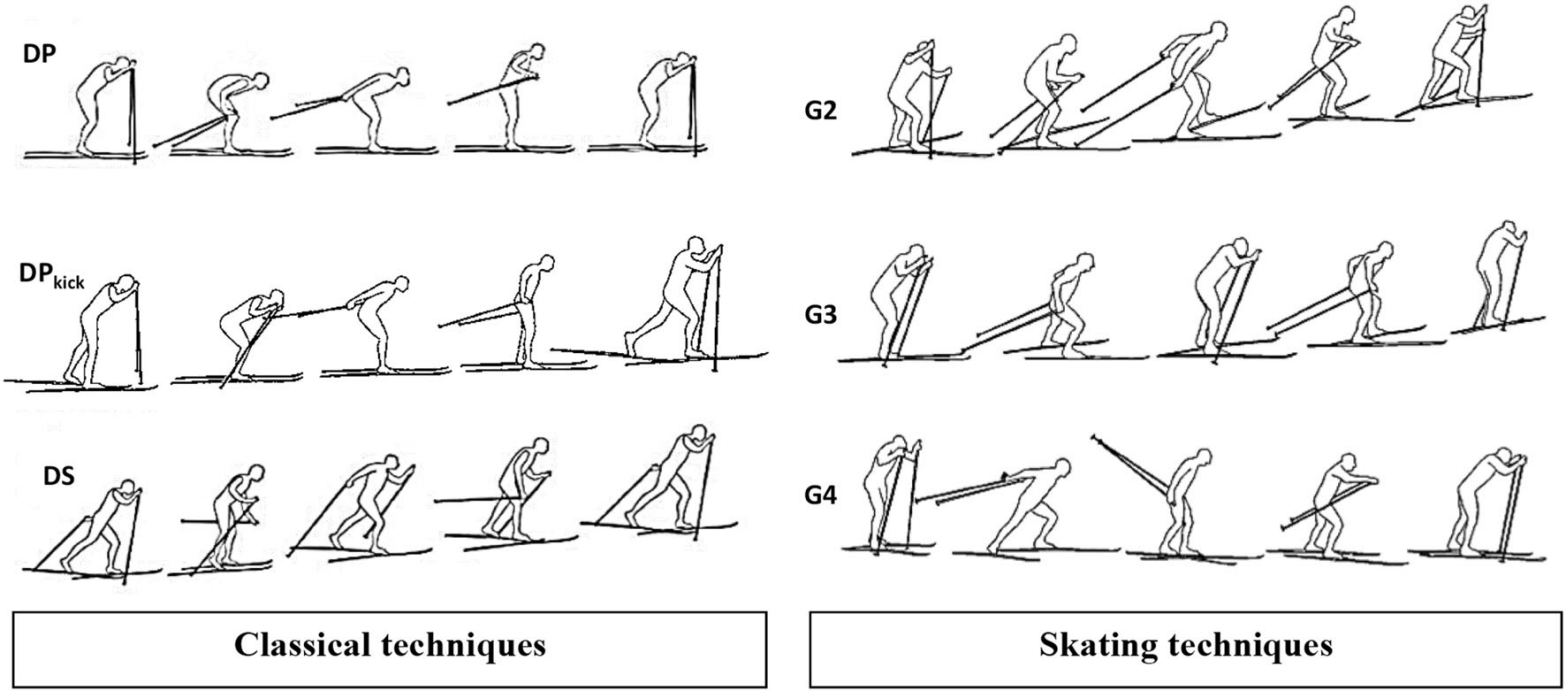
Schematic illustration of a subset of the major classical (*left column*) and skating (*right column*) techniques used in cross-country skiing. *DP* double poling: mainly used on level to moderate uphill terrain. Poles are employed simultaneously with no leg push. *DP*_kick_ kick double poling: mainly used on moderate uphill terrain. Poles are employed simultaneously with one leg push. *DS* diagonal stride: mainly used on moderate to steep uphill terrain. Arms and legs move in a diagonal fashion, with the poling action occurring with the contra-lateral leg push. *G2* gear 2 (or V1 skate): mainly used on moderate to steep uphill terrain. Asymmetric poling action for every second leg push. *G3* gear 3 (or V2 skate): mainly used on level to moderate uphill terrain. One symmetric poling action for each leg push. *G4* gear 4 (or V2 alternate skate): mainly used on level terrain. One symmetric poling action for every second leg push. The double-push skating technique (not illustrated) is a derivative of G3 and involves two pushes with the propulsive leg, rather than one

Data concerning the study aims, population, location, methodologies, key results, and variables examined (as well as their relationships to the performance of elite sprint skiers) were extracted using a standardized form. Each reviewer (CZ and SP) independently extracted data from half of the articles allocated in a randomized fashion. These two reviewers subsequently exchanged articles and the data extracted to verify that the procedure was accurate and complete. The major skiing techniques addressed in the current review are illustrated in Fig. 3. Peak (*V*O_2peak_) and maximal (*V*O_2max_) oxygen uptake reflect different theoretical and practical constructs [27]. However, given the inconsistent usage and interpretation of these terms in the articles reviewed here, the term *V*O_2peak_ is used to encompass both. Similarly, the term peak velocity (*v*_peak_) is applied to encompass parameters referred to as “maximal” or “peak” velocity or speed in these articles. Readers should thus consult the original publications for further specification.

The data were analysed using Microsoft Excel^®^ 2010 (Microsoft Corporation, Redmont, WA, USA). The results were expressed using means and standard deviations (mean ± SD), minimum to maximum ranges, counts, and/or percentages.

## Results

The initial database search yielded 312 hits of which 179 remained after the removal of duplicates. After screening the titles, abstracts, and full-texts, 29 of these articles were found to fulfil the inclusion criteria. Two additional articles were identified through our supplementary searches (Fig. 2).

### Quality Score and Research Design

The quality scores, research designs, subjects, primary variables of interest, and key findings of each article are presented in Table 1. The average quality score of the 31 articles was 66 ± 7 % (range 56–78) on the basis of our modified Downs and Black Quality Assessment Checklist. The main quality issues were failure to consider the representativeness of the study population, state the period during which the subjects were recruited, adjust for confounders, and report actual probability values (e.g., *p* = 0.035 rather than <0.05). Of the 31 studies, 29 were classified as quasi-experimental with a case series design [11, 12, 14, 15, 17–19, 28–48], one as experimental [49], and one as non-experimental [16].

**Table 1:** Summary of the articles reviewed (*n* = 31) with an overview of the subjects and experimental protocols, parameters examined, and major results and implications for the performance of elite sprint cross-country skiers

Study reference, quality, design, and focus	Subjects	Experimental protocol overview	Parameters examined	Major results and implications for sprint skiing performance
Andersson et al. [15] 72 % QE/CS Biomechanics, physiology, and anthropometry	Sample: 9 males Country: Sweden Level: national team Speciality: NS FIS_sprint_: NS	Technique: G3 (V2), DIA, and DP Conditions: laboratory (treadmill, anthropometry) and snow Tests: *V*O_2peak_ with DIA, 20-m sprint with G3 (V2) and DP, 1,425-m TT simulated race skating on snow with ±8.6° incline (one-third flat, one-third uphill, one-thrid downhill), and DXA	20-m speed TT speed (using GNSS) Section speeds (uphill, downhill, flat) Total, lean, and fat mass Number of transitions Techniques used CR *V*O_2peak_ Body composition	20-m speed: DP 7.9 ± 0.4 m/s and G3 (V2) 10.2 ± 0.4 m/s *V*O_2peak_: 73.4 ± 5.8 ml/kg/min 1425-m TT speed: 6.9 ± 0.3 m/s Faster skiers entered uphill sections with greater speed, used G3 (V2) more frequently, and used fewer transitions Slower skiers relied more on G2 (V1) The mean speed on the start section was positively correlated to the total lean mass (*r* = 0.78, *p* < 0.05), but no other correlations between body composition and performance were found Performance is influenced by a range of physiological, biomechanical, and tactical factors
Andersson et al. [40] 61 % QE/CS Biomechanics	Sample: 11 males Country: Norway Level: national team Speciality: 4 sprinters, 4 distance, and 3 all-round skiers FIS_sprint_: 38 ± 21 (sprinters); 97 ± 22 (distance); 85 ± 34 (all-round)	Technique: DIA Conditions: snow Tests: 50-m uphill skiing (7.5° incline) at moderate (65 % intensity: 3.5 ± 0.3 m/s), high (80 % intensity: 4.5 ± 0.4 m/s), and maximal (100 % intensity: 5.6 ± 0.6 m/s) speeds	Speed CR CL CT Pole forces Plantar forces	CR and CL increased from moderate to high speed, while CR was higher and CL lower at maximal than high speed Kick time decreased 26 % from moderate to maximal speed Relative kick and gliding times were altered only at maximal speed, where these were longer and shorter, respectively Rate of force development was enhanced at higher speeds At maximal speed, sprint-specialists were 14 % faster than distance-specialists due to higher CR, peak leg force, and rate of leg force development Pronounced peak leg forces were applied rapidly at all speeds and the relatively shorter gliding and longer kick phases at maximal speed allowed the duration of the kick for force generation to be maintained Rapid generation of leg force is highly important during DIA
Bortolan et al. [44] 61 % QE/CS Biomechanics	Sample: 9 males Country: NS Level: international Speciality: NS FIS_sprint_: NS	Technique: DP Conditions: laboratory (new ergometer) and snow Tests: 50-s DP on the ergometer, maximal 1190-m TT simulated race DP on snow and 3 × 1190-m submaximal TT on snow with the last 180 m all-out (12-min rest between heats)	TT speed Mean power output on the ergometer CT	Mean speed last 180 m of TT: 6.7 ± 0.7 m/s 50-s mean power: 672 ± 167 W (9.2 ± 2.3 W/kg) Performance on ergometer and final 180-m sprint were correlated (*r* = 0.87, *p* < 0.05) CT on the ergometer: 1.1 ± 0.2 s; and field: 0.8 ± 0.1 s The ergometer can be considered to provide ski-specific testing and is useful for evaluating upper-body involvement during skiing in a laboratory setting
Carlsson et al. [43] 72 % QE/CS Physiology and anthropometry	Sample: 10 males Country: Sweden Level: international Speciality: NS FIS_sprint_: 96 ± 27	Technique: DP and DIA Conditions: laboratory (treadmill and ergometer) and snow (actual race) Tests: lactate threshold (4°–8° incline) and maximal treadmill tests (4°–10° incline) with DIA, 60-s DP on a ski ergometer, and 1250-m race prologue on snow using the classical technique	*V*O_2OBLA_ *V*O_2peak_ *V*O_2_ Race speed Anthropometrics	Mean speed race prologue: 6.3 ± 0.1 m/s Race speed was correlated to the absolute values of *V*O_2OBLA_ (*r* = 0.79, *p* = 0.021), *V*O_2peak_ (*r* = 0.86, *p* < 0.001), and *V*O_2_ during DP (*r* = 0.94, *p* < 0.001), as well as body mass (*r* = 0.72, *p* = 0.044) and FIS_sprint_ (*r* = −0.78, *p* = 0.022). However, body mass did not have an influence on the performance models explored Oxygen uptake at different skiing intensities and with different sub-techniques is an indicator of sprint-prologue performance A skier with 1 % higher oxygen uptake is likely to perform 0.2 % better
Carlsson et al. [47] 72 % QE/CS Anthropometry	Sample: 18 males and 16 females Country: Sweden Level: elite Speciality: NS FIS_sprint_: 114 ± 40 (males); 143 ± 48 (females)	Technique: classic (sprint) and skate (distance) Conditions: laboratory (anthropometry) and snow (actual race) Tests: DXA and Swedish National Championships sprint (with classic) and distance (with skate) races on snow	Race times Lean mass, fat mass, and bone Mineral density for the whole body and different body segments	Absolute (in kg) whole-, upper-, and lower-body lean mass, and lower body lean mass were correlated with sprint-prologue performance by both males and females
Carlsson et al. [50] 67 % QE/CS Physiology	Sample: 24 males and 14 females Country: Sweden Level: national and international Speciality: NS FIS_sprint_: 242 ± 105 (males); 242 ± 117 (females)	Technique: DP and DIA Conditions: field (tartan track and asphalt) Tests: 3-km running TT on tartan, 2-km uphill (1.2° incline) roller-skiing TT on asphalt with DP, and 2-km uphill (2.8° incline) roller-skiing TT with DIA on asphalt	TT times FIS points	FIS_sprint_ points and TT times using running, DP, and DIA were correlated for both males and females TT can predict competitive skiing performance by junior cross-country skiers
Losnegard et al. [11] 56 % QE/CS Physiology and biomechanics	Sample: 12 males Country: Norway Level: upper national to international Speciality: sprint, distance, or long-distance skiers FIS_sprint_: NS	Technique: G2 (V1) and G3 (V2) Conditions: laboratory (treadmill) Tests: submaximal tests (4°, 5°, and 6° incline), maximal tests (6°–8° incline), and 600-m self-selected pace (7°) TTs with G2 (V1) and G3 (V2) techniques	TT times *V*O_2peak_ ΣO_2_ demand, ΣO_2_ uptake, and ΣO_2_ deficit Aerobic versus anaerobic contribution to energy production CL CR	Similar 600-m TT performances (~170 s), ΣO_2_ demand, ΣO_2_ uptake, and ΣO_2_ deficit between G2 (V1) and G3 (V2) *V*O_2peak_ with G2 (V1) and G3 (V2) was 72.4 and 71.5 ml/kg/min, respectively, and ΣO_2_ deficit was 62.2 and 60.2 ml/kg ΣO_2_ deficit from the 600-m TT accounted for ~26 % of the total O_2_ cost O_2_ cost at 5°, ΣO_2_ deficit, and *V*O_2peak_ explained 66 to 75 % of the variation in the 600-m TT performance Faster skiers with G3 (V2) showed longer CL but similar CR as slower skiers. With G2 (V1), the contribution of both CR and CL distinguished between skiers with differing 600-m TT times Anaerobic power is a key factor for sprint skiing performance
Losnegard and Hallén [37] 75 % QE/CS Physiology and anthropometry	Sample: 6 males Country: Norway Level: national to international Speciality: 6 sprint and 7 distance skiers FIS_sprint_: 37.3 ± 19.2 (6 sprint skiers); 84.9 ± 32.5 (7 distance skiers)	Technique: G3 (V2) Conditions: laboratory (treadmill) Tests: submaximal test (3.5°–6° incline at 3 m/s), and 1000-m self-selected pace TT (6° incline at 3.25–5 m/s)	Body height, body mass, body mass index *V*O_2peak_ Work economy ΣO_2_ demand, ΣO_2_ uptake, and ΣO_2_ deficit Training history FIS points	Relative *V*O_2peak_ during the TT ranged from 71.8 to 87.8 ml/kg/min ΣO_2_ deficit during the submaximal test ranged from 58.8–91.0 ml/kg Total O_2_ cost (l/min) during the submaximal test was higher in the sprint skiers, but identical between sprint and distance skiers when expressed relative to body mass (ml/kg/min) Absolute *V*O_2peak_ (l/min) and anaerobic capacity (estimated from ΣO_2_ deficit) from the maximal test were higher in sprint skiers, but distance skiers had greater relative *V*O_2peak_ (ml/min/kg) Sprint specialists were heavier and taller than distance specialists Sprint skiers performed more strength and speed workouts than distance skiers
Mikkola et al. [38] 67 % QE/CS Physiology, neuromuscular, and anthropometry	Sample: 16 males Country: Finland Level: international Speciality: NS FIS_sprint_: NS	Technique: DP and G3 (V2) Conditions: laboratory (anthropometry and strength) and field (tartan track) Tests: 30-m peak G3 (V2) and DP speed, 4 × 850-m sprint TTs (20-min rest between heats) with G3 (V2), 10 × 150 m with G3 (V2) (maximal anaerobic skiing test), 2 × 2000 m DP test (submaximal, maximal), strength tests (bench press, trunk flexors, trunk extensors), and estimation of body fat	30-m speed Heat speed in the TT *V*O_2peak_ in each heat Lactate response during each heat Strength Body composition	Mean heat speed was 6.12 ± 0.11 and 5.83 ± 0.15 m/s for fastest and slowest 8 skiers, respectively (*p* < 0.001) Heat speeds did not change during the simulation Relative *V*O_2peak_ (mean: 65.4 ml/min/kg) and peak lactate (mean: 13.3 mmol) during the heats were similar for the groups, but the fastest skiers exhibited higher absolute *V*O_2peak_ (ml/min) Faster skiers had higher speeds during the maximal anaerobic test Relative bench press force was the only neuromuscular variable related to mean speed during the TT (*r* = 0.52, *p* < 0.05) Upper-body and trunk forces correlated to maximal speed and the anaerobic test results Fastest skiers tended to be heavier (*p* = 0.083) during the sprint TT Findings indicate that both anaerobic and aerobic metabolisms are important for sprint skiing on flat terrain under slow conditions Skiers should develop both aerobic and anaerobic capacity, as well as neuromuscular capacities, particularly of the upper body
Mikkola et al. [14] 72 % QE/CS Biomechanics and physiology	Sample: 12 males Country: Finland Level: national and international Speciality: NS FIS_sprint_: NS	Technique: DP Conditions: snow (ski tunnel) Tests: 4 × 1150-m heats (20-min rest between heats) with DP, with first and last 40 m of each heat all-out	Speed Final sprint speed Cycle characteristics Poling forces HR Blood lactate	Speed decreased by 2.7 ± 1.7 % from heat 1 to 4 (6.07–5.92 m/s, *p* = 0.003), as did spurting speed (~16 ± 5 %, *p* < 0.002) Vertical and horizontal poling impulses did not differ significantly within heats, but mean and peak pole forces decreased from start to finish The reduction in speed between and within heats indicated fatigue Fatigue was also indicated by lowered production of pole forces and longer poling times within heats Sprint skiers should improve their resistance to fatigue, particularly in the upper body, to minimize reductions in speed within and between heats
Sandbakk et al. [29] 65 % QE/CS Physiology, biomechanics, and neuromuscular	Sample: 16 males Country: Norway Level: 8 world-class and 8 national level skiers Speciality: sprint FIS_sprint_: 22.5 ± 12.0 (8 world-class); 100.6 ± 45.8 (8 national level)	Technique: G3 (V2) Conditions: laboratory (treadmill, strength) Tests: submaximal test at 14, 16, and 18 km/h (5 % incline), *V*O_2peak_ (8 % incline), *v*_peak_ (8 % incline), and maximal strength (single-leg squat and poling test)	*V*O_2_ Work rate Metabolic rate Gross efficiency HR Blood lactate Time to exhaustion Strength	World-class sprint skiers demonstrated greater gross efficiency than national skiers with G3 World-class and national skiers did not differ in aerobic metabolic rate, but the former showed lower anaerobic metabolic rate World-class skiers achieved higher *v*_peak_ (23.8 vs. 22.0 km/h), higher *V*O_2peak_ (70.6 vs. 65.8 ml/min/kg), and longer times to exhaustion, but had upper- and lower-body strength similar to national skiers World-class skiers used longer CL and lower CR than national skiers at submaximal and maximal speeds World-class skiers were more efficient, perhaps due to better technique and technique-specific generation of power
Sandbakk et al. [13] 70 % QE/CS Physiology, biomechanics, and anthropometry	Sample: 8 males and 8 females Country: Norway Level: World Cup top 30 Speciality: sprint FIS_sprint_: 49.9 ± 12.0 (males); 49.0 ± 14.3 (females)	Technique: G3 (V2) Conditions: laboratory (treadmill) Tests: submaximal test (start 3.9 and 3.6 m/s at 5 % incline for males and females), *V*O_2peak_ (5 % incline), and *v*_peak_ (8 % incline)	VO_2_ Work rate Metabolic rate Gross efficiency HR Blood lactate Time to exhaustion *v*_peak_ CR CL	Larger sex differences in performance and *V*O_2peak_ than reported for comparable endurance sports (higher *V*O_2peak_ and lower percentage body fat in males) At the same submaximal speed, the gross efficiency and work economy of males and females are similar At the same submaximal speed, males used 11 % longer CL at lower CR, as well as 21 % longer CL at peak speed during the *V*O_2peak_ test Males attained a 17 % higher *v*_peak_ and peak treadmill speed (i.e., work rates) during the *V*O_2peak_ (~5 min in duration) and *v*_peak_ (~1 min) tests, respectively
Sandbakk et al. [18] 65 % QE/CS Physiology and neuromuscular	Sample: 16 males Country: Norway Level: 8 world-class and 8 national-level skiers Speciality: sprint FIS_sprint_: 22.5 ± 12 (world-class); 100.6 ± 45.8 (national level)	Technique: G3 (V2) Conditions: laboratory (treadmill) and field (asphalt) Tests: submaximal test (3.9 m/s at 5 % incline on treadmill), *V*O_2peak_ (5 % incline on treadmill), *v*_peak_ (8 % incline on treadmill) 30-m maximal sprint (1 % incline on asphalt), maximal strength (single-leg squat and poling test), training history	*V*O_2_ Work rate Gross efficiency HR Blood lactate Time to exhaustion *v*_peak_ Acceleration Maximal strength FIS points	World-class skiers demonstrated less physiological stress and a higher gross efficiency during the submaximal test World-class skiers showed 8 % higher *V*O_2peak_ and a *V*O_2_-plateau time that was twice as long during the *V*O_2peak_ test World-class skiers showed 8 % higher *v*_peak_, but did not differ from national skiers in acceleration and strength World-class skiers performed 30 % more training, mainly by more low- and moderate-intensity endurance training and speed training Aerobic capacity, efficiency, high-speed capacity, and faster recovery differentiate world- and national-class sprint skiers and might determine sprint performance
Sandbakk et al. [49] 65 % FE/RM Physiology	Sample: 10 males Country: Norway Level: elite junior Speciality: NS FIS_sprint_: NS	Technique: skate Conditions: laboratory (treadmill) and field (asphalt) Tests: pre- and post-intervention (8-week training intervention with increased high-intensity endurance training) tests included 1500-m TT skating (on asphalt) and *V*O_2peak_ test running (10.5 % incline on treadmill)	*V*O_2peak_ *V*O_2_ at VT Work rate HR Blood lactate CR CL Gross efficiency Training history	Aerobic power (*V*O_2peak_ and *V*O_2_ at VT) closely related to sprint performance The intervention group improved sprint performance, *V*O_2peak_, and *V*O_2_ at VT significantly High-intensity endurance training may improve performance and aerobic capacity in junior skiers Sprint skiers should be advised to perform more high-intensity endurance training on level terrain
Sandbakk et al. [17] 78 % QE/CS Biomechanics, physiology, and anthropometry	Sample: 12 males Country: Norway Level: elite Speciality: sprint FIS_sprint_: 44.1 ± 40.0	Technique: skate Conditions: laboratory (treadmill) and snow (FIS sprint skating competition) Tests: 1820-m sprint TT on snow with −6 to +8 % incline (one-third flat, one-third uphill, one-third downhill) and submaximal test (3.9 m/s at 5 % incline), *V*O_2peak_ test (at 5 % incline), and *v*_peak_ test on a treadmill with G3 (V2)	*V*O_2peak_ *v*_peak_ HR Blood lactate TT section speed TT gear selection CL CR Gross efficiency FIS points	TT time was 240 ± 5 s and strongly related to FIS_sprint_ (*r* = 0.96, *p* < 0.001) Mean speed in the final two uphill and final two flat sections correlated strongly with performance Total uphill and flat times were correlated with overall TT time (*r* = 0.91 and 0.82, *p* < 0.001) Relative *V*O_2peak_, *v*_peak_, gross efficiency, and CL were all correlated with TT time (*r* = −0.83 to −0.85, *p* < 0.001) *V*O_2peak_, *v*_peak_, and peak CL in combination provided the best prediction of TT performance (*R* ^2^ = 0.933, *p* < 0.001) High aerobic power is important for sprint TT performance
Stöggl et al. [42] 72 % QE/CS Physiology and biomechanics	Sample: 25 males and 6 females Country: Austria, Slovakia, Switzerland Level: national and student national teams Speciality: NS FIS_sprint_: NS	Technique: DP Conditions: laboratory (treadmill) and field (tartan track and paved road) Tests: 50-m DP *v*_peak_ on track, DP *v*_peak_ on treadmill, 1,000-m DP on treadmill, and 1000-m DP on road (with 1°–4° incline)	50 m-DP time and speed DP *v*_peak_ 1000-m field time 1000-m treadmill time, mean speed, peak speed, and fatigue index (peak minus mean speed)	All tests (50-m DP, DP *v*_peak_, and 1000-m DP on treadmill) were reliable (*r* = 0.78–0.99, *p* < 0.001, CV = 0.79–6.18 %) Time and *v*_peak_ during 50-m DP correlated with *v*_peak_ on the treadmill (*r* = *−*0.90 and 0.86; *p* < 0.001), confirming test validity 1000-m field test time correlated with 1000-m treadmill test time (*r* = 0.96, *p* < 0.01), confirming test validity 50-m DP time, 50-m DP *v*_peak_, treadmill *v*_peak_, and fatigue index all correlated to 1000-m field time (*p* < 0.001) Cross-country DP sprint skiing tests appear to be reliable and valid Developing maximal DP peak speed should improve DP performance over sprint race distances
Stöggl et al. [19] 58 % QE/CS Physiology and biomechanics	Sample: 12 males Country: Austria Level: national team Speciality: NS FIS_sprint_: NS	Technique: classic Conditions: laboratory (treadmill) Tests: DP *v*_peak_, DIA *v*_peak_, *V*O_2peak_, 3 × 1100-m heats simulating a World Cup classic sprint race (25-min and 20-min rest between heats 1 and 2, and heats 2 and 3)	TT time and speed *V*O_2peak_ HR Blood lactate poling frequency CL *v*_peak_	DP *v*_peak_ and DIA *v*_peak_ positively correlated to mean TT speed (*r* = 0.87–0.93, *p* < 0.001) *V*O_2peak_ test time (*r* = 0.74, *p* < 0.01), but not *V*O_2peak_ value, significantly correlated to sprint performance *V*O_2_ and tidal volume decreased from heat 1–3 Faster skiers generated significantly higher blood levels of lactate Faster skiers used fewer pole plants and diagonal cycles, as well as longer CL, thereby achieving more propulsion with equal CR A statistically non-significant tendency was found for the best-performing skiers trying to use DP-kick in the moderate uphill sections and when changing grades, while skiers of moderate performance seldom used DP-kick The positive influence of maximal speed on sprint performance suggests that increasing the proportion of training designed to improve speed might be beneficial for all skiing techniques
Stöggl et al. [46] 58 % QE/CS Biomechanics and physiology	Sample: 25 males and 6 females Country: Austria, Slovakia, Switzerland Level: national and student national team Speciality: NS FIS_sprint_: NS	Technique: DP Conditions: laboratory (rollerboard ergometer and treadmill) and field (tartan track) Tests: two-phase test on a rollerboard, with a four-repetition maximal test and 40-repetition test; DP 50-m *v*_peak_ on track, DP *v*_peak_ on treadmill, 1000-m DP on treadmill	50 m-DP time and speed DP *v*_peak_ 1000-m treadmill time, mean speed, peak speed, and fatigue index (peak minus mean speed) mean and peak speed, time to Rollerboard test peak speed, peak acceleration, mean power, peak force, time to peak force, rate of force development, impulse, and fatigue indexes (peak minus mean values)	Four-repetition maximal and 40-repetition speed and power values were reliable Mean peak speed during 40 repetitions and power-based fatigue indexes exhibited the best correlation with 1000-m DP speed Peak speed during the four-repetition maximal test accounted for 84 % of the variation in 50-m DP performance Peak speed and power during the four-repetition maximal accounted for 61 % of the variation in the 1000-m DP speed, and peak speed during the 40-repetition test accounted for 69 % of the 1000-m DP speed The four-repetition maximal test alone is simple, reliable, and valid for diagnosing upper-body and DP performance in skiers
Stöggl et al. [32] 61 % QE/CS Biomechanics	Sample:13 (sex not indicated) Country: Austria Level: national and student teams Speciality: NS FIS_sprint_: NS	Technique: G3 (V2) and double push Conditions: snow Tests: 100-m sprint on 2° uphill	Speed Pole force Plantar force Knee angle EMG CL CR CT	Double push was 2.9 ± 2.2 % faster than G3 (*p* > 0.001) Double push involved longer CL and CT, and lower CR Peak knee angle, range of knee extension, angular knee speed, plantar force, and muscle activity during the first push-off are greater with double push No difference in pole force between these two techniques Double push can be employed during cross-country skiing to improve the speed of short maximal sprints on moderately uphill slopes
Stöggl and Müller [33] 68 % QE/CS Biomechanics, physiology, and anthropometry	Sample: 24 males Country: Austria, Greece Level: national team Speciality: NS FIS_sprint_: NS	Technique: DP, DIA, and G3 (V2) Conditions: laboratory (treadmill) Tests: MART protocol performed using the DP (at 1.5° incline), DIA (at 4.5 m/s), and G3 (V2, at 2.5° incline) techniques	*v*_peak_ HR Blood lactate and glucose CR CL CT Anthropometry (body height and pole length)	At MART termination, peak speed was 8.17 ± 0.3 and 8.9 ± 0.3 m/s during DP and G3 (V2), and peak grade was 11° ± 1° during DIA MART protocol is transferable to all three skiing techniques With all techniques, skiers elevated speed by increasing CR and attempting to maintain CL 13 skiers switched to the double-push technique during the G3 (V2) test and reached higher maximal speeds DP exhibited an optimal CL (~at 7.5 m) and CR (~at 1.2 Hz) at *v*_peak_ Duration of the swing phase was most closely related to performance, where the duration of the arm swing positively correlated with performance in all techniques Peak lactate level correlated to *v*_peak_ with all techniques Absolute body height and pole length correlated to peak DP speed only, indicating a tendency for taller skiers to be faster
Stöggl et al. [31] 58 % QE/CS Biomechanics	Sample: 6 males Country: Austria Level: national and student national team Speciality: sprint FIS_sprint_: NS	Technique: G2 (V1), G3 (V2), and double-push Conditions: snow Tests: 60-m uphill (7–10° incline) at maximal speed	Sprint time and speed Pole force Plantar force Knee angle CR CL CT	60-m speed with G2 (V1), double-push, and G3 (V2) was 5.51 ± 0.23, 5.44 ± 0.23, and 5.21 ± 0.25, m/s Speed with G2 (V1) and double-push techniques was similar and both faster (~5.5 and 4.3 %) than G3 (V2) Double push and G3 (V2) involved longer CL and CT, lower CR, shorter duration of the first push-off, and longer flight time than G2 (V1). Peak plantar and impulse forces during the second push-off were also higher with comparable poling frequencies and forces CL, peak plantar force, and knee extension range of motion and angular speeds are higher in double-push than G3 (V2) In comparison with G2 (V1), double-push requires less space due to less lateral displacement and no technique transitions upon entering and leaving an uphill section
Stöggl et al. [30] 59 % QE/CS Anthropometry	Sample:14 males Country: Sweden, Austria, Norway Level: national and international Speciality: sprint FIS_sprint_: NS	Technique: DIA and DP Conditions: laboratory (anthropometry and treadmill) Tests: DXA scan, *v*_peak_ with DIA (at 7° incline) and DP (at 1° incline)	DP and DIA *v*_peak_ Total, lean, fat, and bone mass for the whole body, trunk, legs, and arms Body dimensions (segment lengths)	*v*_peak_ with DP and DIA was 31.8 ± 1.9 and 18.4 ± 0.8 km/h Height and most other body dimensions were unrelated to *v*_peak_ Body, total trunk, and lean trunk mass strongly related to DP *v*_peak_ Absolute and relative body and trunk mass related to DIA *v*_peak_ Skiers should focus on increasing whole body lean mass for improving *v*_peak_, particularly of the trunk for DP and of the trunk and arms for DIA
Stöggl et al. [12] 58 % QE/CS Biomechanics and neuromuscular	Sample: 16 males Country: Sweden, Austria, Norway Level: national and international Speciality: sprint FIS_sprint_: NS	Technique: DP, DIA and G3 (V2) Conditions: laboratory (treadmill and strength) Tests: strength and power tests, *v*_peak_ during DP (at 1° incline), DIA (at 7° incline), and G3 (V2, at 2.5° incline)	DP, DIA, and G3 (V2) *v*_peak_ CR CL CT Pole force Plantar force Upper- and lower-body strength and power tests (isometric leg tests, squat jump, bench press, bench-pull, and brutal-bench repetitions)	Relationships between exercises involving general strength and *v*_peak_ depend on the skiing technique None of the isometric strength tests were related to *v*_peak_ Number of brutal-bench repetitions, bench press and pull power, and squat jump force measures related to DP *v*_peak_ Bench press and pull, and squat jump measures related to DIA *v*_peak_ 1-repetition maximum bench press (in kg) and squat jump height (in m) related to G3 (V2) *v*_peak_ With all three techniques, increase to *v*_peak_ involved enhanced CR, with an associated reduction in CL during DP and DIA With all three techniques, strategies utilised when approaching *v*_peak_ differed between faster and slower skiers Faster skiers not only applied greater forces, but also displayed better temporal coordination of force application Sprint skiers need a certain level of strength, but more appears not necessarily superior
Stöggl and Holmberg [45] 67 % QE/CS Biomechanics	Sample: 16 males Country: Sweden, Austria, Norway Level: national and international Speciality: sprint FIS_sprint_: NS	Technique: DP Conditions: laboratory (treadmill and strength) Tests: *v*_peak_ during DP (at 1° incline)	Pole force 3D kinematics	*v*_peak_ during DP was 31.7 ± 1.7 km/h Relative (% body height), but not absolute, pole length related to *v*_peak_ Faster skiers exhibit a distinct preparation phase to the pole plant, with the duration of the preparation phase predicting DP *v*_peak_ Faster skiers exhibited longer CL and absolute swing and poling times, as well as greater peak pole forces that occurred later in the poling phase
Stöggl and Holmberg [41] 58 % QE/CS Biomechanics	Sample: 15 males Country: Sweden, Austria, Norway Level: national and international Speciality: sprint FIS_sprint_: NS	Technique: G2 (V1) Conditions: laboratory (treadmill) Tests: *v*_peak_ and submaximal speeds (13, 14, 15, 16 km/h at 7° incline)	*v*_peak_ Pole forces Plantar forces 3D kinematics CT CL CR	*v*_peak_ during G2 (V1) was 17.8 ± 0.8 km/h As speed increased, CR elevated by 20 %, whereas poling and leg push-off times fell by 21 % Poling time was shorter, propulsive pole impulse forces lower, and leg push-off time longer on the “weak” than “strong” sides of the body Power in the direction of skiing rose with increasing speed Poles generated ~44 % of the total propulsion, being more effective than legs (~59 % vs. 11 %, *p* < 0.001) Faster skiers exhibited more well-synchronized poling and more symmetric edging and force generation by legs, as well as more effectively transforming resultant forces into propulsion CL was unrelated to both *v*_peak_ and total propulsive force impulses Certain differences in the pole and leg forces on the “strong” and “weak” sides of the body were pronounced, highlighting asymmetry of the G2 (V1) technique
Tønnessen et al. [48] 72 % QE/CS Physiology	Sample: 66 males and 45 females Country: Norway Level: international Speciality: 59 sprint or distance, 33 biathlon, and 19 Nordic combined FIS_sprint_: NS	Technique: not applicable Conditions: laboratory (treadmill) Tests: MART	*V*O_2peak_ History of medal at the Olympics or World Championships	On average, Olympic-medal benchmarks for relative *VO*_*2*peak_ values were 78 and 68 ml/kg/min for male and female sprint skiers, respectively. The corresponding benchmarks for absolute *V*O_2peak_ values were 6.3 and 4.0 l/min The differences in relative and absolute *V*O_2peak_ values between medallist and non-medallist sprint skiers were trivial High *V*O_2peak_ is necessary for high-level sprint skiing performance
Vesterinen et al. [39] 58 % QE/CS Physiology, biomechanics, and neuromuscular	Sample: 16 males Country: Finland Level: national team Speciality: sprint FIS_sprint_: NS	Technique: G3 (V2) Conditions: field (tartan track) Tests: 30-m *v*_peak_, 4 × 850-m heats with first and last 50-m all-out (20- min rest between heats)	*v*_peak_ *V*O_2peak_ HR Blood lactate EMG CT CL CR	Time and mean speed did not change during the 4 heats, but initial speed in heat 4 was slower Peak *V*O_2_, HR, and lactate did not change from heat 1 to 4 Maximal speed within heats decreased from start to end, as did muscle activity and CR Changes in metabolic responses, cycle variables, *v*_peak_, and muscle activity within each heat indicated induction of fatigue Correlation between peak lactate and speed during heat 1 indicated that anaerobic power was especially important during this first heat Mean *V*O_2peak_ correlated with change in speed from heat 1 to 4, indicating that skiers with more aerobic power developed less fatigue during the simulation
Zory et al. [16] 56 % NE/CS Biomechanics	Sample: 30 males Country: NS Level: World Cup Speciality: sprint FIS_sprint_: NS	Technique: DIA Conditions: snow (video analysis of the Viessmann World Cup 1.2 km classic race) Tests: final 200 m (5 % incline) filmed and analysed	Race speed Stride speed Stride length Stride rate	Mean race speed was 7.33 m/s Mean stride speed, length, and rate were 4.78 m/s, 2.16 m, and 2.2 Hz, respectively Stride speed correlated with race speed and stride rate Faster skiers used higher stride rate Speed on the uphill section analysed had an important impact on race outcome Skiers need to develop high frequencies to attain high speeds
Zory et al. [34] 78 % QE/CS Biomechanics and physiology	Sample: 7 males Country: Italy Level: national team Speciality: sprint FIS_sprint_: NS	Technique: classic Conditions: snow and laboratory (ergometer) Tests: 50-s maximal DP ergometer test, voluntary and evoked knee flexor and extensor MVC, 3 × 1200-m TT using the classic technique with the last 180 m all-out with DP (12-min rest between heats)	TT time and speed Blood lactate Knee flexor and extensor MVC EMG Ergormeter force, velocity, and power	Mean speed was similar for all heats (~6.97 m/s), but the final sprint speed was significantly lower in heat 3 than in heat 1 (6.55 vs. 6.13 m/s) Lactate increased significantly from heat 1 to 3 Knee extensor MVC was 9.8 ± 9.5 % lower post TT, with no significant difference in knee flexor MVC Mean power frequency of rectus and biceps femoris muscles was significantly lower after the TT Upper-body force and power were reduced after the TT Changes were indicative of fatigue induced by the TT protocol
Zory et al. [36] 63 % QE/CS Biomechanics and neuromuscular	Sample: 8 males Country: Italy Level: national team Speciality: sprint FIS_sprint_: NS	Technique: classic Conditions: snow and laboratory (strength) Tests: 50-s maximal DP ergometer test, knee flexor and extensor MVC, 3 × 1200-m TT (12-min rest between heats) using the classic technique with flat, uphill, and downhill sections. The last 180 m of the TT was all-out with DP (at 2 % incline)	TT time and speed Blood lactate Knee flexor and extensor MVC EMG Ergometer force, velocity, and power CL CR Cycle speed Ankle, knee, hip, trunk, elbow and pole angles	Mean speed was similar for all heats (~6.66 m/s), but the final sprint speed was significantly lower in heat 3 than 1 (6.57 vs. 6.23 m/s) Lactate increased significantly from heat 1 to 3 Knee extensor MVC was 10.4 ± 10.4 % lower post TT, with no significant difference in knee flexor MVC Mean power frequency of rectus and biceps femoris muscles was significantly lower after the TT Upper-body force and power were reduced after the TT Cycle speed decreased in successive heats Joint and poling angles were generally similar in all heats, except for the trunk, hip, and poles being less flexed at the end of the poling phase in heat 3 than in heat 1, suggesting less effective force application
Zory et al. [35] 76 % QE/CS Neuromuscular, biomechanics, and physiology	Sample: 8 males Country: NS Level: international Speciality: sprint FIS_sprint_: NS	Technique: classic Conditions: snow Tests: 3 × 1200-m TT (12-min rest between heats) using the classic technique with flat, uphill, and downhill sections. The last 180 m of the TT was all-out with DP (at 2 % incline)	TT time and speed EMG- activation and frequency Blood lactate	Final sprint speed was significantly lower in heat 3 than in heat 1 (6.41 vs. 5.98 m/s) Lactate increased significantly from heat 1 to 3 Activation patterns were maintained, but 6 of 8 muscles exhibited signs of fatigue The biceps brachii muscle exhibited the greatest fatigability Fatigue was more pronounced in the upper than lower body The higher speed in heat 1 than in heat 3 was not explained by changes in muscle activation

*CL* cycle length, *CR* cycle rate, *CS* case series, *CT* cycle time, *CV* coefficient of variation, *DIA* diagonal stride, *DP* double poling, *DXA* dual-energy X-ray absorptiometry, *EMG* electromyography, *FE* fully-experimental, *FIS* International Ski Federation, *FIS*_*sprint*_ International Ski Federation sprint points, *GNSS* global navigation satellite system, *HR* heart rate, *MART* maximal anaerobic running test, *MVC* maximal voluntary contraction, *NE* non-experimental, *NS* not stated, *ΣO*_*2*_ accumulated oxygen, *OBLA* onset of blood lactate, *QE* quasi-experimental, *RM* repeated measures, *TT* time-trial, *v*_*peak*_ peak velocity, *VO*_*2peak*_ peak oxygen uptake, *VT* ventilatory threshold

### Subjects and Experimental Protocols

The mean number of subjects was 18 ± 19 (range 6–111), including altogether 567 subjects from eight different countries (Table 1). Most subjects (74 %) were male, 17 % were female, and the sex of the remaining 9 % involved in three articles [16, 32, 36] was not specified. The mean age of subjects across studies (weighted by articles’ sample size) was 24.6 ± 2.7 y. Only nine articles explicitly reported the FIS-sprint points earned by their skiers, with a weighted mean of 116 ± 78 [13, 17, 18, 29, 37, 40, 43, 47, 50]. As shown in Table 1, not all subjects were sprint-skiing specialists.

Fifteen articles (48 %) focused on the classic technique [14, 16, 19, 30, 34–36, 40, 42–47, 50], 11 (35 %) on the skating technique [11, 17, 18, 28, 29, 31, 32, 37, 39, 41, 49], and the remaining five (16 %) involved both [12, 15, 33, 38, 48]. Seventeen studies (55 %) involved roller-skiing on a treadmill in the laboratory [11–13, 15, 17–19, 29, 30, 33, 37, 41–43, 45, 46, 49], and 12 (39 %) examined skiing performance on snow [14–17, 31, 32, 34–36, 40, 44, 47]. Only two of these studies (6 %) evaluated skiing in both of these environments [15, 17]. Other approaches and environments to assessing ski-specific skills involved ergometers [34–36, 43, 44, 46], tartan tracks [38, 39, 42, 46, 50], and paved roads [18, 42, 50].

Of the various experimental protocols employed to assess sprint cross-country skiing performance (see “Task” in Table 1), simulated races (i.e., time trial) involving either a single or repeated heats were chosen in numerous studies [14, 15, 17, 19, 34–39, 42–44, 46, 49] and actual races in two [16, 47]. Performance was heterogeneously defined across the literature, being based on a single heat time-trial [15, 17, 43, 49], a repeated heats time-trial [19, 34–36, 38, 39], peak skiing speed [12, 15, 30–33, 41, 45, 46], or level of expertise [18, 29, 48] (e.g., World Class skiers vs. national-level skiers and medallists vs. non-medallists). Only nine studies (29 %) examined the relationship between FIS-sprint points and experimental variables [15, 17, 18, 29, 37, 40, 43, 47, 50].

### Performance Factors

A range of factors were found to influence or be related to elite sprint cross-country skiing performance (see “Key Results and Implications for Performance” in Table 1). In most cases, a combination of physiological and biomechanical aspects were evaluated (see “Focus” in Table 1), although 14 studies were considered to focus more on biomechanics [12, 14–17, 31–33, 36, 40, 41, 44–46], 13 on physiology [11, 13, 18, 19, 29, 37–39, 42, 43, 48–50], two on anthropometrics [30, 47], and two on neuromuscular characteristics [34, 35]. The key factors identified are summarized in Table 1, outlined here, and addressed in greater detail in Sect. 4.

With respect to physiology, aerobic [17, 18, 38, 39, 48, 49] and anaerobic capacities [11, 19, 37–39], as well as skiing economy and efficiency [17, 18, 28, 29] were found to be major factors that distinguish sprint skiers with different levels of performance. Most findings indicate that aerobic capacity (i.e., *V*O_2peak_) exerts a significant impact on performance during time-trials involving both a single and repeated heats [17, 29, 37, 38, 43, 49]. Vesterinen et al. [39] stressed the importance of aerobic characteristics in sprint cross-country skiing as well, showing that high level aerobic characteristics prevented fatigue accumulation during a simulation of a cross-country sprint skiing competition (four 850-m repeated heats with 20-min recovery between heats on roller-skis using the G3 (V2) skate technique), whereby individuals with greater mean *V*O_2peak_ demonstrated smaller changes in mean velocities from heat 1 to 4. However, it should be noted that although aerobic capacity was higher in world-class than national-level skiers [18], there was no meaningful difference in the relative and absolute *V*O_2peak_ values of world-class skiers who won medals at the Olympics or World Championships and those who did not [48]. Anaerobic capacity is also a key indicator [11, 19, 29, 37–39], particularly with respect to performance during the first of repeated heats [39]. In addition, the more rapid reduction in blood levels of lactate in world-class sprint skiers following a single 4- to 6-min roller-skiing test to exhaustion on a treadmill using the G3 (V2) skate technique indicates that faster recovery might be beneficial in connection with the high-intensity repeated heats and limited recovery time characteristic of sprint skiing [18].

Among the biomechanical analyses carried out on a variety of techniques (see “Discipline” in Table 1), maximal speed [12–14, 17, 19, 29, 31, 33, 40–42, 44], cycle characteristics [11–17, 19, 29, 31, 33, 36, 39–41, 44, 45], and kinetics (pole and plantar forces) [12, 14, 31, 32, 40, 41, 45] were the variables most often examined. Other biomechanical aspects addressed were joint angles [31, 32, 36, 41], electromyography [32, 34–36, 39], and performance measures using specialized ergometers or tests [44, 46].

In most cases, at top speed, the cycle length reached a plateau or even shortened [12, 33, 40], with increased speed across various techniques relying primarily on elevations in cycle rates [12, 16, 33, 40]. In general, faster skiers demonstrated longer cycle lengths at peak, racing, and submaximal speeds [13, 17, 19, 33, 45]; longer swing (recovery) times [12, 33, 45]; better temporal coordination, including timing of the application of force [12, 45]; and greater effectiveness in transforming resultant pole and leg forces into propulsive ones [41].

Analysis of a single heat of a sprint race, both simulated and real, revealed that performance on the uphill sections exerted the greatest influence on race outcomes [15–17], with strong correlations between the time spent on these sections and the total heat time [15, 17]. Gear selection and transition are also important aspects, with faster skiers making fewer transitions during a single heat of a skating race and making greater use of the G3 (V2) than the G2 (V1) technique [15] since they could employ, for instance, G3 (V2) also when on steep inclines rather than reverting to G2 (V1). During 1100 m of classical sprint skiing, faster skiers were observed to employ fewer overall cycles of movement and fewer cycles of diagonal skiing, as well as tending to use the kick double poling technique more frequently [19].

Regarding anthropometry, the relative amount of lean mass has been associated with sprint double poling [14, 30, 38], diagonal stride [30], prologue race [47], and heat start [15] performances. More specifically, the absolute expression of lean mass in the whole, upper (arms and trunk), and lower body has been correlated with sprint-prologue performance in one study [47]. However, in another study, the total body mass and trunk lean mass were positively related to both double poling and diagonal stride peak speed [30], with the upper and lower body lean masses only contributing to diagonal stride peak speed. Although certain researchers have reported a relationship between body mass (kg) [30, 38, 43] and height [33] to selected measures of sprint skiing performance, others have found no such association [15, 30].

Several articles described upper-body power and strength as determinants of classic sprint performance in time-trials involving repeated heats and double poling peak speed on snow [34, 44], with skiers with greater double poling speeds producing a greater amount of upper-body power [44, 46]. Measures of dynamic strength (e.g., squat jump height and bench press power) have also been related to varying extents to peak speed in connection with double poling, diagonal stride, and G3 (V2) technique [12, 38], as have maximal isometric trunk flexion and extension [38].

Fatigue has been examined using electromyography, speed profiles, biomechanical measures (e.g., poling forces, cycle characteristics, and fatigue indices), and physiological measures (e.g., lactate, heart rate, and *V*O_2_) in several studies in connection with classical sprint cross-country ski racing [14, 19, 34–36, 38], but only in one study in connection with skating [39]. In the latter investigation, muscle activity was attenuated at the end of each heat, although fatigue did not appear to accumulate across the four heats examined, which exhibited similar speed profiles [39]. In connection with sprint skiing using the classical technique, Zory and colleagues [34, 36] observed maintenance of mean speed in successive heats during a simulated race, although several parameters indicated fatigue, primarily of peripheral origin. For instance, lower- and upper-body force and power were impaired [34, 36], the mean power frequencies of electromyographic signals were reduced [34–36], and the twitch contractile properties of muscles were altered [34].

## Discussion

Despite the relatively recent addition of sprint cross-country skiing events to the Winter Olympic Games, 31 original research articles were found to investigate factors relating to performance in this sport at the time of this systematic review. Clearly, performance in sprint cross-country skiing is complex and multi-factorial, as demonstrated by the large proportion of these articles that addressed physiological, biomechanical, anthropometric, and neuromuscular aspects simultaneously.

To summarize, adequate aerobic and anaerobic capacity are essential for successful sprint cross-country skiing. Although both systems play a role throughout the sprint competition, the anaerobic system may contribute more to the first of repeated heats and the aerobic system more to the subsequent heats [38, 39]. Generation of high speed via an optimization of the interaction between cycle rate and length [12, 29, 40] is of utmost importance [17–19, 28–30, 42]. Faster sprint skiers employ different strategies to approach maximal speeds, relying on technique [12, 33], movement efficiency [13, 17, 19, 33], and coordinated movement patterns and force application [12, 33]. During races, performance on uphill sections exerts the greatest influence on the final outcome [16, 17] given that skiing speed decreases the most on steep uphill sections [15]. Hence, strong uphill performance is critical. Although a certain level of strength is required for successful sprint skiing, more does not necessarily appear to translate into superior sprint skiing performance [12, 18, 29]. Once the required strength level is reached, developing ski-specific power has the potential to influence performance to a greater extent, and can improve work efficiency, power output, and selected physiological parameters in well-trained skiers [51].

To elaborate, training strength and endurance capacities, particularly of the upper body, have the potential to reduce the negative impact of fatigue during sprint races involving a single or repeated heats [14, 34, 35], although leg [12, 40] and trunk [12, 30, 38] strength should clearly not be neglected. Heavy strength training is widely used by elite sprint skiers, and several studies have demonstrated correlations between measures of strength and various indicators of sprint skiing performance [12, 38, 46]. At the same time, beneficial effects of strength training interventions on sprint skiing performance are not always seen [52, 53]. A recent intervention involving heavy strength training by junior female cross-country skiers (published after our systematic search) resulted in no significant effect on submaximal O_2_ cost during double poling or on average power output during maximal double-poling effort on an ergometer for 20-s or 3-min intervals [52]. Similarly, Losnegard et al. [53] observed no significant effect of heavy strength training on roller-skiing peak speed or single-heat time-trial performance by well-trained senior and junior Norwegian skiers.

The technical aspects of cross-country skiing are highly complex and, although increases in strength might improve sprint skiing performance, the timing of force application and the development of ski-specific power may exert a more pronounced influence than maximal strength per se [45, 51]. At the same time, it is important to note that in both studies cited above [52, 53], heavy strength training had no negative impact on the performance measures investigated, produced similar or even higher gains in these measures than traditional training, and was implemented for only 10–12 weeks, which might be insufficient for noticeable adaptation that influence skiing performance specifically. Furthermore, the effects of heavy strength training on performance during actual sprint competitions were not investigated.

Finally, available evidence regarding the impact of anthropometric characteristics on sprint skiing performance and peak speed is mixed; for instance, both beneficial [30, 33, 38, 43] and inconsequential [15, 30] effects of body height, mass, and body dimensions have been reported. However, more lean mass has been related to better outcomes during the first section of a single-heat time-trial performed on snow [15], during time-trials involving four 850-m heats roller-skiing on a tartan track [38], and peak roller-skiing speed on a treadmill [30]; and, therefore, skiers should strive to optimize this particular anthropometric characteristic.

### Physiology

One of the earliest publications in this field introduced a valid and reliable test concept for assessing sprint skiers, namely that of short-duration maximal double-poling roller-skiing efforts to predict double-poling sprint performance both in and outside of the laboratory [42]. Similar approaches were applied in several of the other studies included in this review [12, 13, 15, 18, 19, 30, 41, 45, 46], many highlighting that maximal speed over short distances (20–50 m) or relatively short durations (~60 to 90 s) utilizing the various techniques [double poling, G3 (V2), and diagonal stride] correlated well with performance during time-trials involving single or repeated heats and/or FIS-sprint points [15, 18, 19]. Later, Sandbakk et al. [29] introduced a submaximal incremental roller-skiing test on a treadmill to quantify gross efficiency and aerobic/anaerobic metabolic rates, and an incremental time-to-exhaustion test to assess peak oxygen uptake in sprint skiers. More recently, the relative contribution of the aerobic and anaerobic energy systems to performance have been studied using ski-skating sprint time-trials at a self-selected pace first by Losnegard et al. [11], and more recently in the classical technique with junior cross-country skiers by McGawley et al. [54].

From the studies herein reviewed, the physiological factors observed to differ the most between sprint skiers of varying performance levels are aerobic capacity [17, 18, 38, 39, 49], anaerobic capacity [11, 19, 37–39], and skiing economy and efficiency [17, 18, 28, 29]. Many of these studies indicate that *V*O_2peak_ exerts a significant impact on performance [17, 29, 37, 38, 43, 49], with higher *V*O_2peak_ (as assessed during roller-skiing on a treadmill) being associated with better FIS-point rankings [15], on-snow sprint skiing performance during a simulated prologue (classic and skate techniques) race [17, 43], and speed maintenance uphill during the later stages of a single-heat simulated sprint race using the skating technique [15, 17]. Carlsson et al. [43] suggested that sprint race performance improves by 0.2 % for each 1 % increase in absolute *V*O_2peak_, although this simplified estimation does not account for all the factors that impact race performance. Furthermore, skiers with higher recorded *V*O_2peak_ during a simulated sprint cross-country skate competition on a tartan track [using the G3 (V2) technique] were better able to maintain speed during four successive heats, indicating that aerobic power was especially important in the later heats [39]. However, it should be noted that once athletes reach a certain level of performance, such as competing in the Olympics or World Championships, higher aerobic capacity does not ensure a place on the winners’ podium [48].

The anaerobic capacity of sprint skiers is also a key performance indicator [11, 19, 29, 37–39], with this system estimated to contribute ~22 to 26 % of the total O_2_ demand during a 600-m sprint skiing time-trial lasting ~170 to 190 s [11, 54]. In other words, the anaerobic system appears to contribute ~25 % of the total energy required during a sprint-skiing heat. However, during sections of a sprint-skiing heat, the O_2_ demand can be much higher, implying an even greater anaerobic contribution [11, 17]. During maximal testing of anaerobic capacity using G3 (V2) involving four 850-m repeated heats on snow, faster sprint skiers have been shown to attain higher speeds [38], with world-class sprint skiers demonstrating greater gross efficiency and lower anaerobic metabolic rates at submaximal speeds compared to national-level sprint skiers using the same technique [18, 29]. Furthermore, sprint cross-country skiers are reported to have higher anaerobic capacities (i.e., greater accumulated oxygen deficits) than distance skiers [37], again suggesting the key role played by this capacity in elite sprint skiing. Other physiological characteristics related to indicators of sprint skiing performance include oxygen uptake at the lactate [43, 49] and ventilatory [49] thresholds.

It is worth noting here that the methods utilized to quantify the anaerobic contribution to sprint cross-country skiing differ. Earlier studies focused on increases in [17, 18] and peak [19] blood levels of lactate, whereas more recently the maximal accumulated oxygen deficit has been characterized [11, 37, 54–57]. Blood levels of lactate depend on the production, release, and utilization of this compound by active muscles. The arms of elite skiers are reported to release more lactate than they utilize during submaximal roller-skiing efforts with the classical technique, while the opposite situation has been observed in the legs [58]. Thus, the relative involvement of the arms and legs in cross-country skiing will exert a considerable influence on the blood levels of lactate, rendering the use of this measure to quantify the anaerobic capacity of skiers less valid.

Although no studies pertaining directly to recovery met inclusion for review here, an athlete’s ability to recover is an aspect worth addressing in sprint cross-country skiing given the repeated-heat format of competitions (4 × 3- to 4-min efforts within a 2- to 3-h time span). Race analyses and practical observations of cross-country skiing competitions indicate that although some skiers are very good during the first half of the prologue, they cannot sustain their level of performance throughout the remainder of the competition (unpublished observation). Recently, a few studies have addressed the effects of varying recovery modes on repeated efforts in the context of sprint skiing [55, 59]. Although active recovery was associated with a slightly, but significantly, greater effect on aerobic turnover than passive recovery [55], roller-skiing performance (two 800-m heats on a treadmill) using the G3 (V2) technique was similar. On the other hand, Stöggl and colleagues [59] observed that passive recovery resulted in greater decrements during high intensity runs to exhaustion compared to active recovery strategies implemented with or without supplementation. In combination, these two studies suggests a benefit of active versus passive recovery strategies during sprint skiing competitions, although further research is obviously needed to determine the optimal dosage and explore alternative recovery strategies.

The only fully-experimental study that met the criteria for inclusion in this review involved an 8-week intervention designed to reveal the effects of increased high-intensity endurance training (i.e., 2.5-fold increase in long duration high-intensity intervals performed at 85–92 % of peak heart rate) on sprint-skiing performance and aerobic characteristics of elite junior skiers [49]. The high-intensity endurance training positively impacted performance of a 1.5-km time-trial (~3.5 min) performed on roller skis outdoors in the skating technique, *V*O_2peak_, and oxygen uptake at the ventilatory threshold [49]. Hence, a greater proportion of high-intensity endurance training on level terrain was recommended as a means of improving the performance of young athletes. However, it is difficult at this point to confirm whether such gains would also be achieved by senior athletes. The overall paucity of fully-experimental studies included for review here reflects the inherent difficulties associated with studying high-level athletes and instigating interventions within their training programs. Training studies are time-consuming and require serial assessments and timely follow-ups [60].

### Biomechanics

Studying cross-country skiing biomechanics is intricate given that the involvement of the upper and lower body differs across techniques and depends on terrain. For instance, the relative contribution of the upper body to propulsive forces during double poling is higher than with the other techniques. Across all techniques, high skiing speeds are reported to stem from an optimization of cycle rate and length [12, 29, 40]. Sandbakk et al. [29] observed that as skiing speed with the G3 (V2) technique increased to peak, skiers increased both cycle rate and length [29]. However, most researchers report a plateau or decrease in cycle length at maximal speeds [12, 33, 40, 61], with elevated speed relying primarily on more rapid cycle rates [12, 16, 33, 40, 61]. Nonetheless, skiers who demonstrated longer cycle lengths at given cycle rates outperformed those skiers with shorter cycles during both classic and skate skiing [12, 13, 17, 19, 33, 45]. The poling and swing time of the arms are also reported to decrease with increasing speed during G3 (V2), double poling, and diagonal stride [12, 33], with faster skiers employing longer swing times and spending a greater proportion of their cycle in the swing (recovery) than the thrust phase [12, 33, 45].

In the case of sprint competitions in classical cross-country skiing, top-ranked skiers have been observed to employ the double poling technique exclusively in all heats up to the finals when the track profile is appropriate [45]. With regards to double poling, biomechanical studies indicate that the duration of the preparation phase strongly relates to peak speed on flat terrain, with faster skiers exhibiting greater cycle lengths, longer swing and poling times, later and higher peak pole forces, and smaller poling angles with respect to the vertical direction at pole plant [45]. Noteworthy here is that the biomechanical and physiological aspects of double poling on uphill terrain at high skiing speeds were first investigated only recently, with double poling uphill being associated with much shorter swing times, as well as greater, later, and more effective pole forces than on flat terrain [62]. Within and between heats of a simulated sprint competition conducted on snow, fatigue was manifested by a decrease in double poling speeds [14, 36], increase in poling time [14], and decrease in poling force [14]. Stöggl and Müller [33] also observed more rapid cycle rates and shorter cycle times when double poling in a fatigued state at the end of a maximal anaerobic test, but no such changes when using the diagonal stride technique. Moreover, these investigators noted that absolute poling times employing these two classical techniques were maintained with fatigue, but that relative poling times became longer and swing times became shorter. Leg thrust times also increased with fatigue when utilizing the diagonal stride. In summary, faster sprint skiers appear to demonstrate greater maximal propulsive pole forces, as well as more resistance to, and better maintenance of, poling technique in connection with fatigue.

The techniques of cross-country skate skiing have evolved markedly over the years, with more explosive sub-techniques being developed and utilized successfully for relatively short periods of time [7, 32]. For instance, although G2 (V1) has been shown to be faster than G3 (V2) on steep uphill inclines [31], on less steep inclines (2°–10°), higher speeds can be reached using the double-push sub-technique of G3 (V2) [12, 31–33]. Higher-ranked skiers have also been observed to rely on the G3 (V2) technique on uphill sections of a single-heat time-trial simulation to a greater extent than those ranked lower. This difference is thought to reflect more frequent use of the double-push technique, higher uphill speed enabling use of a higher gear, and the superior upper-body strength and resilience to fatigue required for using G3 uphill exhibited by the better skiers [15]. That said, individual differences must always be taken into consideration since, at an individual level, certain athletes have been shown to achieve faster, slower, or comparable peak uphill speeds using the G2 (V1) compared to the double-push technique [31]. The double-push technique is very demanding (e.g., requires greater muscular activity of key muscles and plantar forces), which might restrict its use to short sections or for fast tactical accelerations during sprint events [32].

When approaching peak speed, faster skiers exhibit biomechanical strategies that differ from those of slower skiers [12, 32, 33, 45], not only in the magnitude of forces applied, but also with respect to their temporal coordination and instances of application [12]. For example, at peak G2 (V1) speed, faster skiers performed more synchronous pole plants, exhibited greater effectiveness in transforming resultant into propulsive forces, and used narrower edging angles [41]. They also generated greater propulsion at equal poling frequencies while employing the classic technique [19], and longer cycle lengths during G3 (V2) [13, 17, 29]. These findings agree with Holmberg et al. [63] who proposed more than a decade ago that faster skiers utilize a more sprinter-like double-poling technique, with higher peak poling forces and impulses, shorter relative poling times, and longer relative recovery times. Although contemporary double poling technique has evolved, these relationships are still evident during double poling on flat terrain [62]. Stöggl and colleagues [12] demonstrated that at maximal skiing speeds, the time for propulsion by the poles was ~200 ms during double poling and G3 (V2), with as little as 150 ms for the leg push-off during diagonal stride [40]. These values are similar to the very short contact times associated with jumping exercises (e.g., the ground contact time during a drop jump), emphasizing the necessity for rapid production of force. At present, intervention studies concerning different techniques are lacking. Whether interventions aimed at modifying technical aspects can enhance the peak speed and sprint race performance of elite skiers remains to be determined scientifically.

### Neuromuscular Factors

In cross-country skiing, the upper body plays a crucial role in overall performance [34, 44], and has been reported to contribute considerably to propulsion in several techniques [40, 41]. Custom-made ski-specific upper-body ergometers and assessment protocols have shown that faster skiers produce greater upper-body power [44, 46], indicating the significance of explosive upper-body strength. Furthermore, skiers with higher upper-body power and double poling peak speed have demonstrated less fatigue during a single-heat 1000-m double poling sprint test [46]. Still, during simulated time-trials involving three 1200-m heats performed on snow, electromyography [34, 35] and kinetics [14] data have indicated greater upper body than lower body muscle fatigue [35]. Therefore, training the fatigue resistance of the upper body is also to be recommended and integrated into training programs of elite sprint skiers [14, 35].

The ability to develop large peak leg forces rapidly is also of fundamental importance to maximizing skiing speed (shown in particular for the diagonal stride) [12, 40], in agreement with previous cross-country skiing research not specifically addressing sprint skiing [64, 65]. Peak leg forces during diagonal stride on snow are reported to reach almost twice body mass and, at maximal speed, to be developed quite rapidly (~100 ms) [40]. Like sprint running [66], high forces (relative to the body mass of an individual) must be generated during short contact times and training of dynamic strength and motor skills designed to improve this ability could be beneficial to sprint skiers.

Previous studies have revealed that isometric upper- and lower-body strength do not correlate well with peak speed during the double poling, diagonal stride, and G3 (V2) techniques [12]; that dynamic strength (power output and vertical jump performance) correlates particularly well with peak speed during double poling and diagonal stride [12]; and that the 1-repetition maximal upper- and lower-body strength of national- and international-level sprint skiers do not differ [18, 29]. Overall, these findings indicate that: (1) elite skiers attain necessary levels of maximal strength beyond which further improvement does not necessarily enhance performance; (2) dynamic strength is a better indicator of performance than static strength and should be utilized in connection to training; and (3) repeated high-intensity efforts might be more suitable for assessing sprint-skiing abilities than a single maximal effort. There is also evidence that the trunk muscles contribute to the development of high speed [12, 30, 38], i.e., skiers with stronger [38] and leaner [30] trunks and who performed a greater number of brutal-bench repetitions [12] were also faster using the double poling, diagonal stride, and G3 (V2) techniques.

### Anthropometry

Modifiable anthropometric characteristics, such as muscle mass and relative lean mass, have been related to the peak speed attained by elite cross-country skiers [30]. Lean mass in particular has been correlated with indicators of sprint performance (e.g., peak speed and single-heat time-trial) with both the classic and skating techniques [15, 30, 38, 47]. Absolute whole-, lower-, and upper-body lean mass (in kg) show large to very large correlations with sprint-prologue performance in both men and women (*r* = − 0.66 to −0.82, *p* < 0.05) [47]. Despite indications that total body mass (kg) [30, 38, 43] and height [33] relate to sprint cross-country skiing performance, with elite sprint skiers reported to be being taller and heavier than distance skiers [17], other findings have found no such associations [15, 30]. Perhaps more lean mass simply reflects greater muscle mass and strength, thereby corroborating earlier findings on cross-country skiing not related specifically to sprint events [67].

### Other Considerations

The present review and articles included have several limitations. Given the array of experimental tasks and different types of skiers involved, it is challenging to generalize the findings from one study to another. In addition, although much focus has been placed here on studies that utilized peak speed as the performance outcome, this is only one of the parameters related to performance. As is the case with maximal strength, greater peak speed does not necessarily result in better sprint-skiing performance, especially when peak speed is determined over a very short distance [18]. Several other factors must also be considered.

FIS points are used to rank skiers internationally, but less than a third of the publications reviewed here reported FIS points [13, 17, 18, 29, 37, 40, 43, 47, 50] or attempted to correlate them with the investigated performance outcomes [15, 17, 18, 29, 37, 40, 43, 47, 50]. Also, sprint cross-country skiing competitions have evolved since their introduction to the World Cup circuit. Initially, four skiers competed head-to-head on a relatively flat course for ~2 min. Nowadays, six skiers compete against one another on a longer and hillier course, with races typically lasting ~3 min. These changes now enable endurance skiers to perform more successfully in sprint events also, particularly female skiers where sprint specialization is less evident [28].

Moreover, individual differences were noted in several of the articles reviewed [33, 35, 36]. As mentioned above, certain elite skiers showed faster, slower, or comparable peak speeds on uphill terrain using the G2 (V1) versus double-push technique [31]. Furthermore, individual responses to fatigue during a simulated classical sprint race were also observed in national skiers, with certain of these athletes decreasing both cycle length and rate or reducing only one of these two factors [36]. Pacing and tactical strategies have also been reported to differ between skiers and can impact race outcomes [15, 17], with most skiers seen to adopt a positive pacing strategy (i.e., athlete’s speed progressively declines during the race [68]). More studies of individual responses to repeated heats, as well as of racing tactics associated with successful competition outcomes are required.

Factors associated with skiing performance with one technique or on one type of terrain do not necessarily exert an impact with other techniques or on different types of terrain. For instance, the factors associated with double poling performance on flat terrain are not the same as those associated with double-poling performance uphill [62]. The relative involvement of upper and lower body differ across techniques, where the propulsive forces are primarily developed from the upper body during double poling and from both the upper and lower body during the diagonal stride and skating techniques G2, G3, and G4 (V1, V2, and V2 alternate). Furthermore, although there are some indications that performance in the laboratory provides a valid indication of skiing performance on snow [13, 44], conclusions drawn from roller-skiing on a treadmill or on asphalt, paved roads, and tartan tracks might not always apply to on-snow skiing.

In addition, elite cross-country skiers and trainers should bear in mind that several factors other than those reviewed here, such as recovery [55] and nutritional strategies [69], may influence repeated sprint skiing performance. The muscle fibre composition or genetic make-up of elite sprint cross-country skiers has not yet been examined. These factors would be of considerable interest since: anaerobic performance has been related directly to the proportion of type II muscle fibres [70]; sprint runners are reported to exhibit a greater proportion of fast-twitch muscle fibres [71]; and genetic factors have been shown to strongly influence the ability of skeletal muscles to produce explosive forces [72].

The age of peak cross-country sprint performance is another question yet to be addressed [73]. Work by Allen and Hopkins [73] indicates that for a sprint race of ~3 min in duration, the optimal age is around 22.5 y (±1.3), but this needs to be confirmed for sprint skiers. Lastly, as in research on elite alpine ski racing [23], a relatively low number of female skiers were included in the studies reviewed here, which is of concern in light of the sex differences identified in elite sprint cross-country skiers with respect to physiology [13], biomechanics [13], anthropometry [47], degree of specialization [28], and factors predicting performance [47, 50]. Clearly, further studies involving internationally competitive female sprint cross-country skiers are highly recommended, and such reports have now begun to appear more frequently since the date of our systematic search [52, 74, 75]. Fatigue and recovery during repeated heats tactics, and sex differences should be the focus of future studies in this field.

## Conclusions

Cross-country skiing is demonstrably a demanding and complex sport. Successful sprint skiing requires numerous physiological, biomechanical, anthropometric, and neuromuscular attributes, including well-developed aerobic and anaerobic capacities, effective biomechanical techniques, a high proportion of lean mass, and the capability to generate high forces rapidly. The ability to attain high speed at the start of a sprint race, at any given point when required (e.g., when being challenged by a competitor), and in the final section of each heat, despite fatigue, is crucial to sprint skiing performance. A certain level of strength is also required, as well as the ability to tolerate fatigue during competitions and recover between heats. To expand our understanding of elite sprint skiing performance, future research should further investigate performance on snow, repeated-heat sprint performance, optimal recovery strategies, and the elite female skier, as well as factors not yet researched, such as muscle fibre constitution, genetic factors, nutrition/supplementation, and age of peak performance.
